# Immune-Neuroendocrine Interactions and Autoimmune Diseases

**DOI:** 10.1080/17402520600877059

**Published:** 2006

**Authors:** Luis J. Jara, Carmen Navarro, Gabriela Medina, Olga Vera-Lastra, Francisco Blanco

**Affiliations:** 1 Research Division Clinical and Epidemiology Research Unit and Internal Medicine Department Hospital de Especialidades Centro Médico La Raza, IMSS Mexico City Mexico; 2 Universidad Nacional Autónoma de México Mexico City Mexico; 3 Clinical Research Direction Molecular Biology Laboratory Instituto Nacional de Enfermedades Respiratorias Mexico City Mexico; 4 Immunology Research Unit Hospital de Pediatría Centro Médico Nacional Siglo XXI, IMSS Mexico City Mexico

## Abstract

The relationship between immune-neuroendocrine system is firmly established. The messengers of this connection are hormones, neuropeptides, neurotransmitters and cytokines. The immune-neuroendocrine system have the capacity to synthesize and release these molecules, which, in turn, can stimulate or suppress the activity of immune or neuroendocrine cells by binding to receptors. In fact, hormones, neuropeptides and neurotransmitters participate in innate and adaptive immune response.

Autoimmune rheumatic diseases (ARD) are characterized by aberrant production of pro-inflammatory cytokines, which are a potent activator of the HPA axis. In consequence, high levels of pro-inflammatory hormones such as estrogens and prolactin, and low levels of glucocorticoids, an anti-inflammatory hormone, have been described in the active phase of ARD. In addition, high levels of pro-inflammatory hormones and cytokines have also been frequently detected in organ involvement of patients with ARD, suggesting an abnormal local neuroendocrine immune interaction. There is evidence that hormonal changes may appear before the symptomatic phase of the disease. Therefore, it is possible that a pro-inflammatory hormone favors the rupture of tolerance, which is a key feature of autoimmune diseases. The interactions between the immune-neuroendocrine system have a major impact on our understanding of the pathogenic mechanisms, diagnosis and therapy of ARD.


*Clinical & Developmental Immunology*, June–December 2006; 13(2–4): 109–123

### Immune-neuroendocrine interactions and autoimmune diseases

LUIS J. JARA[1,2], CARMEN NAVARRO[3], GABRIELA MEDINA[1], OLGA VERA-LASTRA[1,2], & FRANCISCO BLANCO[4]

[1]*Research Division, Clinical and Epidemiology Research Unit and Internal Medicine Department, Hospital de Especialidades Centro Médico La Raza, IMSS, Mexico City, Mexico,* [2]*Universidad Nacional Autónoma de México, Mexico City, Mexico,* [3]*Clinical Research Direction, Molecular Biology Laboratory, Instituto Nacional de Enfermedades Respiratorias, Mexico City, Mexico, and* [4]*Immunology Research Unit, Hospital de Pediatría, Centro Médico Nacional Siglo XXI, IMSS, Mexico City, Mexico*

**Abstract**

The relationship between immune-neuroendocrine system is firmly established. The messengers of this connection are hormones, neuropeptides, neurotransmitters and cytokines. The immune-neuroendocrine system have the capacity to synthesize and release these molecules, which, in turn, can stimulate or suppress the activity of immune or neuroendocrine cells by binding to receptors. In fact, hormones, neuropeptides and neurotransmitters participate in innate and adaptive immune response.

Autoimmune rheumatic diseases (ARD) are characterized by aberrant production of pro-inflammatory cytokines, which are a potent activator of the HPA axis. In consequence, high levels of pro-inflammatory hormones such as estrogens and prolactin, and low levels of glucocorticoids, an anti-inflammatory hormone, have been described in the active phase of ARD. In addition, high levels of pro-inflammatory hormones and cytokines have also been frequently detected in organ involvement of patients with ARD, suggesting an abnormal local neuroendocrine immune interaction. There is evidence that hormonal changes may appear before the symptomatic phase of the disease. Therefore, it is possible that a pro-inflammatory hormone favors the rupture of tolerance, which is a key feature of autoimmune diseases. The interactions between the immune-neuroendocrine system have a major impact on our understanding of the pathogenic mechanisms, diagnosis and therapy of ARD.

**Keywords:** *Immune-neuroendocrine, hormones, neuropeptides, neurotransmitters, autoimmune rheumatic diseases*

The relationship between neuroendocrine and the immune system has been analysed since the 1980s, opening new important horizons in the neuroendocrine-immune field (Weigent and Blalock 1987). It has been known that the neuroendocrine system can both directly and indirectly influence the developmental and functional activity of the immune system. On the other hand, the immune system can collaborate in the regulation of endocrine activity. The network of connections is mediated by nerve pathways, hormonal circuits, cytokines, neuropeptides and chemokines (Szyper-Kravitz et al. 2005). Hormones and neuropeptides are secreted not only by endocrine glands but also by many extra gland sites including the immune cells, and these molecules can stimulate or suppress the immune cells by binding to receptors (Besedovsky and del Rey 1996). Changes in hormonal levels in autoimmune rheumatic diseases (ARD), could depend on the enhancement of coordinated bi-directional communications between neuroendocrine and the immune systems. High levels of pro-inflammatory hormones such as estrogens (E) and prolactin (PRL), and low levels of glucocorticoids (GC), an anti-inflammatory hormone, have been described in the active phase of systemic lupus erythematosus (SLE), rheumatoid arthritis (RA) and other autoimmune diseases. High levels of E and PRL can increase IFN gamma and IL-2 by Th1

Correspondence: L. J. Jara, Hospital de Especialidades Centro Médico La Raza, IMSS, Seris/Zaachila s/n, colonia La Raza, cp. 02990, Mexico City, Mexico. Tel: 52 55 5724 5900. Ext. 23015. E-mail: luis_jara_quezada@hotmail.com

ISSN 1740-2522 print/ISSN 1740-2530 online © 2006 Taylor & Francis
DOI: 10.1080/17402520600877059

lymphocytes activation and autoantibody production through Th2 lymphocytes activation (Lang 2004; De Bellis et al. 2005). High levels of pro-inflammatory hormones and cytokines have also been frequently detected in organ involvement of patients with ARD, suggesting an abnormal local neuroendocrine immune interaction (Straub and Cutolo 2001; Elenkov et al. 2005). Of particular interest is the possibility that a pro-inflammatory hormone favors the rupture of tolerance, which is a key feature of autoimmune diseases. Furthermore, hormonal changes may appear before symptomatic phase of the disease (Walker and Jacobson 2000; Peeva and Zouali 2005). The purpose of this review is to analyse the role of the immune neuroendocrine system on inflammation and autoimmune diseases and their interactions during pregnancy.

### Inflammation. immune response and neuroendocrine system

The nervous, endocrine and immune systems orchestrate a complex and integrated response to stimuli through interaction of molecules to maintain the homeostasis (Eskandari et al. 2003). Immune, endocrine or neural cells can both synthesize and express receptors for cytokines, hormones, neurotransmitters and neuropeptides. These products act in an autocrine, paracrine and endocrine manner working as bidirectional feedback communication mediators (Besedovsky and del Rey 1996). In fact, hormones, neuropeptides and neurotransmitters participate in innate and adaptive immune response.

### Innate immunity and neuroendocrine system

### *Hypothalamic-pituitary-adrenal axis. HPA*

The innate immunity, the first line of immune defense, involves the mononuclear phagocyte cells that process antigens and become the primary antigen-presenting cells (APCs). APCs express on their surface Toll-like receptors (TLRs) which recognize certain pathogen-associated molecular patterns (PAMPs) found in pathogens. TLRs–PAMPs engagement transmits transmembrane signals that activate the nuclear factor (NF-kB) and mitogen activated protein kinase (MAPK) pathways, with transcription of genes encoding cytokines. The innate immune system had others resources to recognize microbial pathogen which include natural killer (NK) cells, $\gamma\delta$ T cells, complement system and type I Interferons (IFNs) (Medzhitov and Janeway 2000; Hansson et al. 2002; Hoebe et al. 2004). Systemic pro-inflammatory cytokines such as IL-1$\alpha$/$\beta$ and IL-6 synthesized by APC through TLRs pathways, across barriers of the central nervous system (CNS) to activate in the circumventricular organs (CVO), and cells forming the blood-brain barrier. The organum vasculosum of the laminae terminalis (OVLT) is a region involved in mechanisms of thermoregulation. Neurons of the OLVT and the paraventricular nucleus (PVN) control the corticotrophin-releasing hormone (CRH) release (Turrin and Rivest 2004). Besides the hypothalamic-pituitary-adrenal (HPA) axis activation, cytokines induce a change in the mood, behavior and pain responses (Eskandari et al. 2003; Turrin and Rivest 2004; Chida et al. 2005). Therefore, the release

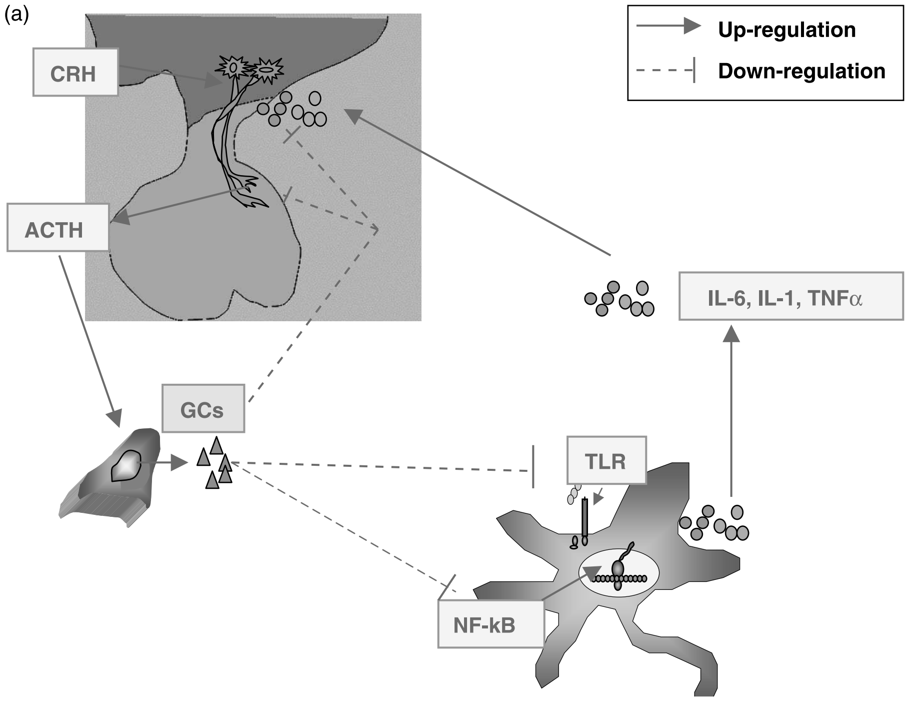

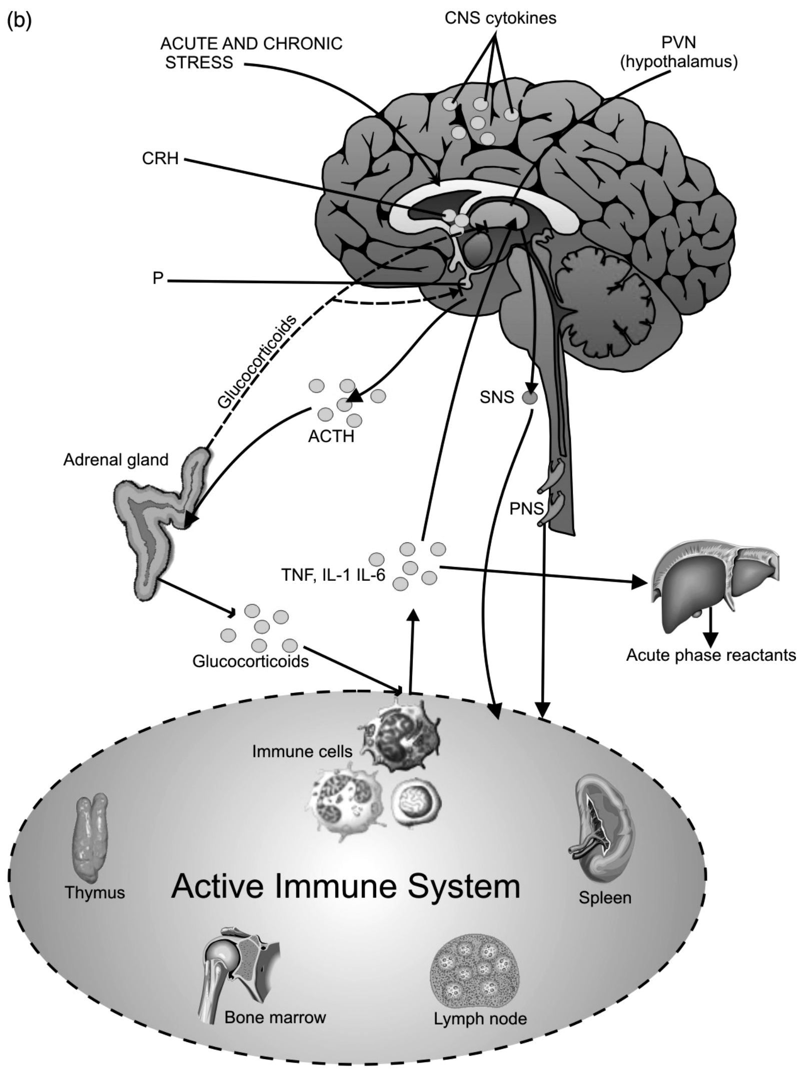

Figure 1. Immune-neuroendocrine system. During inflammatory process, active immune system synthesize and release pro-inflammatory cytokines that stimulate the liver to produce acute phase reactants and activation of CNS that leads to the release of CRH and other neuropeptides by the hypothalamus. CRH increase ACTH production by the pituitary gland, which increase secretion of GC. The vagus nerve and sympathetic nervous system are other systemic routes to maintain immune-neuroendocrine conections. Figure modified from Nat Rev Immunol 2006; 6: 318–328 (reference 16). Toll like receptors (TLR) with PAMPs engagement transmits transmembrane signals that activate the nuclear factor (NF-kB) with transcription of genes encoding cytokines. IL-1, TNF $\alpha$ and IL-6 across barriers of the CNS, stimulate neurons of PVN with activation of the HPA axis. The release of glucocorticoids (GC) is the most powerful endogenous mechanisms to suppress the inflammatory response genes and the Toll receptor signaling cytokines.

of glucocorticoids (GCs) is the most powerful endogenous mechanisms to suppress the inflammatory response genes and the Toll receptor signaling (Medzhitov and Janeway 2000; Turrin and Rivest 2004) (Figure 1).

GCs exert their immunomodulatory effects through the glucocorticoid receptor (GR) localized in the cytoplasm and complexed to heat-shock proteins (inactive form). The binding of GCs to their intracellular receptors causes the release of the complex. The complex steroid/receptors moves to the nucleus and binds to specific DNA sequences to regulate gene transcription interfering with NF-kB and activator protein-1 (AP-1) (Sternberg 2006). Recent evidences suggest that GR represses a set of functionally related inflammatory responses genes by disrupting p65/interferon regulatory factor (IRF) complexes required for TLR4- or TLR-9 (Ogawa et al. 2005).

The complement system contributes significantly to the innate immune response. The receptor C3a (C3aR) is present on adrenal gland, pituitary, glial cells and neurons. Francis et al. (2003), hypothesized that C3a and C3adesArg may activate the HPA axis to release GC, Growth hormone (GH), PRL and ACTH. CRH is up-regulated by serotonergic, cholinergic and catecholaminergic systems. Other neuropeptides such as $\gamma$-aminobutyric acid/benzodiazepines (GABA/BZB) inhibit CRH secretion (Eskandari et al. 2003).

### Adaptive immune and neuroendocrine systems

*Hypothalamic-pituitary-adrenal axis. HPA*

GC inhibit the production of pro-inflammatory cytokines and metalloproteinases and stimulate the production of anti-inflammatory cytokines shifting the Th1/Th2 balance towards the Th2 phenotype, which stimulates the humoral immunity. Th2 cytokines are angiostatic and maintain the integrity of the microvascular system by preserving permeability and inhibiting adhesion molecules expression limiting endothelial activation and cell migration to inflammatory foci (Chikanza and Grossman 2000; Straub and Cutolo 2001).

*Hypothalamic-pituitary-gonadal axis*

Females have greater responsiveness of both antibody- and cell-mediated immune than male. Sex steroids have binding sites in primary lymphoid organs and peripheral immune cells, suggesting both Gonadotrophin-releasing hormone (GnRH) and sex steroids have a role in immune system, acting through a local autocrine way and/or through hypothalamic-pituitary-gonadal (HPG) axis activation. Lymphoid organs and peripheral immune cells express GnRH and their receptors (GnRH-R). GnRH is involved in thymus maturation and has a potent immune-stimulating action increasing the levels of IL-2R, IFN-γ and T helper cells (Tanriverdi et al. 2003).

Estrogens (E) and androgens (A) influence immune cell development in lymphoid tissue and have an immunomodulatory effect on T and B cells in adult life. Estrogen receptor (ER) is identified in mature peripheral B and T lymphocytes, in contrast with androgen receptors (ARs) which are absent (Tanriverdi et al. 2003). Androgens influence the size and composition of the thymus, and have a direct or indirect effect on enhance suppressor effect increasing CD4− CD8+ cells. Testosterone (T) inhibits IL-1β secretion by peripheral blood mononuclear cells (PBMCs). Dihydrotestosterone (DHT) represses the expression and activity of the human IL-6 gene. It is unclear the effects of A on peripheral B cells, but it seems to diminish the production of autoantibodies (Tanriverdi et al. 2003). E is an important stimulator of humoral immunity and they act in both bone-marrow and peripheral B cells, and the levels of circulating antibodies (Ab), by increasing IL-10 production. E has effect on T cells, stimulates and increased CD4+CD8− and CD4+CD8+ cells, and it can activate an extra-thymic pathway of autoreactive T cell differentiation. Physiologic concentrations of E increase the pro-inflammatory cytokine production and pharmacologic concentrations decrease cytokines synthesis (Straub and Cutolo 2001; Eskandari et al. 2003; Tanriverdi et al. 2003).

*Hypothalamic-pituitary-thyroid axis*

Thyroid stimulating hormone (TSH) is a central component of the hypothalamic-pituitary-thyroid (HPT) axis. Immune cells can be able to produce biological active TSH. Wang and Klein (2001) proposed that TSH produced by immune cells during stress situation, plays a dual role consisting on TSH communication with immune system and serving to regulate homeostasis by modulating thyroid hormone activity. Immune cells have receptors for thyrotrophic and thyroid hormones (Cremaschi et al. 2000). It has been observed an increase of T3 and T4 levels after immunization. *In vivo* treatment with T4 increases alloantibodies titer during the early state of alloimmunization, while treatment with propylthiouracil down regulates the humoral response (Klecha et al. 2000). The opposed is observed in chronic stress where a reduction in serum levels of T3, was found. These findings suggest that acute and chronic stress induce an alteration of the HPT axis function that modified the adaptive immune response.

*Prolactin and immune system*

PRL is a 23 Kda polypeptide hormone produced by the anterior pituitary gland that stimulates mammary growth and differentiation. PRL is stimulated by suckling and stress and is inhibited by hypothalamic dopamine. This hormone is produced in a number of sites outside the pituitary, including the brain and immune cells. PRL is important in maintaining immune competence and has a role in the pathogenesis of ARD. All activities of PRL are mediated by the PRL-receptor, a member of the hematopoietin cytokine receptor superfamily (Walker and Jacobson 2000; Szyper-Kravitz et al. 2005). PRL expression and PRL-receptors have been demonstrated in immune cells suggesting that the hormone may act in an auto- or paracrine way. PRL stimulates inducible nitric oxide (NO) syntheses production, Ig release and cytokine expression in human leukocytes (Lahat et al. 1993; Matera et al. 1999; Matalka 2003). PRL is a T cell mitogen through the PRL-R/Janus activating kinase (JAK)/Stat/IRF-1 signaling pathway and NF-kB signals (Yu-Lee 2002). T lymphocytes PRL expression is subject to regulation by cytokines. Both IL-2 and IL-4 reduced PRL mRNA levels in T lymphocytes (Gerlo et al. 2005). PRL significantly enhanced the expression of CD69, CD25 and CD154 and cytokines secretion, and modulate B cells development (Morales et al. 1999; Chavez-Rueda et al. 2005; Takizawa et al. 2005). Therefore, PRL has an important role in adaptive immune response.

After inflammatory challenge an immediately raise of serum GC, epinephrine and norepinephrine is observed. However, a prolonged increased in the activity of the HPA axis conduce to a prompt lost of the anti-inflammatory mediators with increase of pro-inflammatory mediators such as PRL, 17-β-estradiol (E2) and substance P (Straub and Besedovsky 2003).

The concept of an integrated bidirectional regulated neuroendocrine-immune adaptive response to stress has strong experimental support (Wilder 1995). In this regard, Ezzat et al. (2005), identified expression of Ikaros transcription factors (a differentiation factor of leukocytes lineage), in mouse pituitary cells and demonstrated a direct role for Ikaros in the regulation of ACTH expression and adrenocortical hormone production. Ikaros influence the maturation of the fetal pituitary corticotrophin and the secretion of ACTH before and after birth indicating that it is an integrator of the stress and immune systems, and their defects may produce endocrine and/or immune diseases (Chrousos and Kino 2005).

### Hypothalamic pituitary adrenal axis and autoimmune rheumatic diseases

### *Rheumatoid arthritis*

RA is mediated by high levels of pro-inflammatory cytokines, which are a potent activator of the HPA axis. In consequence, the endogenous GCs produced by the adrenal glands, have an important role in the inflammation cascade in RA (Morand and Leech 2001).

*Animal models*. The classic studies of Sternberg et al. (1989) using a model with Lewis and Fisher strain of rats (the former is inflammatory-susceptible and the latter is resistant), demonstrated that in Lewis rats, susceptibility to inflammatory disease is related to blunted HPA axis response, whereas Fisher rats have exaggerated response. Recently, this hypothesis was challenged. Chover-Gonzalez et al. (2000) observed that in high-stress situations, rats with a greater corticosterone response to the acute stress developed early and more severe arthritis than rats with a less profound corticosterone response, suggesting that the balance of pro- and anti-inflammatory factors released in response to stress may influence the progress of adjuvant arthritis (AA). In another study, Lewis rats were divided in high-active (HA) and low-active (LA) animals based on their exploratory activity in the open field. The AA activity in the open field was associated with the severity of bone destruction, low production of bone-protective cytokines (IL-10 and IFN-γ), and high levels of vascular endothelial growth factor (VEGF), leading to more synovial proliferation in LA animals. Of interest, there were no differences between HA and LA rats in corticosterone response after acute or chronic immune challenge (Sajti et al. 2004).

*Human studies*. The most persuasive evidence was provided by the observation that clinical signs of inflammation in RA could be exacerbated by the administration of metyrapone, an 11β-hydroxylase inhibitor that inhibits adrenal GC synthesis (Morand and Leech 2001). Lost of cortisol circadian rhythm has been reported in patients with high activity RA (Neeck et al. 1990). Chikanza et al. (1992) demonstrated in RA patients a failure to increase cortisol secretion following surgery, despite high levels of interleukin-1 β (IL-1β) and IL-6. These patients had a defective HPA response, as evidenced by a diurnal cortisol rhythm of secretion which was at the lower limit of normal, in contrast to those patients with osteomyelitis. CRH stimulation test in RA patients showed normal results, suggesting a hypothalamic defect. The key question that remains unanswered is whether the HPA-axis abnormalities seen in humans with RA are a predisposing factor or a consequence of the disease. The examination of case reports, such as those of RA onset after treatment of Cushing's disease, leads to a strong suspicion of HPA axis function as a barrier to the development of RA, which once breached permits this disease to ensue (Uthman and Senecal 1995; Straub and Cutolo 2001). Significant dissociation of serum cortisol and dehydroepiandrosterone sulfate (DHEAS) levels was found in the subgroup of asymptomatic premenopausal women who developed RA before age 50 (Masi et al. 2000; Masi et al. 2005). Gutierrez et al. (1999) showed that active RA is associated with subtle dysfunction of the HPA and normal PRL secretion. Using the insulin tolerance test, RA patients have decreased plasma cortisol levels compared to healthy controls, despite elevated levels of IL-6. The defect is probably located at the adrenal level and may be of pathogenic significance for the development of RA (Eijsbouts et al. 2005).

One way for the study of immune neuroendocrine interactions is through specific membrane receptor for GC (mGR) on RA lymphocytes. Measurement of mGR has produced contrasting results from slightly elevated to decreased numbers of GRs in PBMCs in RA (Schlaghecke et al. 1994; Bartholome et al. 2004). Neeck et al. (2002) using immunoblot analysis to detect mGR in lymphocytes of untreated RA patients, GC-treated RA patients and healthy controls found that untreated RA patients exhibited a significantly higher amount of mGR, whereas GC-treated RA patients showed a strongly decreased receptor density. These results suggest a functional dysregulation of the HPA axis. mGCR are localized not only on inflammatory lymphoid cells of RA patients, but also on synoviocytes, suggesting that

GC could act directly on these cells (Tohyama et al. 2005).

*Systemic lupus erythematosus*

*Animal models*. Lechner et al. (1996) analysed the HPA axis response to recombinant human (rhu) IL-1α in lupus-prone mice. This model produced nearly the same levels of plasma corticosterone after injection of rhu-IL-1alpha as seen in normal mice, but had baseline corticosterone levels consistently higher (Lechner et al. 1996). MRL/MP-fas (Lpr) mice not only show an altered GC response mediated via HPA axis, but are also affected by disturbance of GC and melatonine circadian rhythms. These results confirm the existence of a disturbed immunoendocrine communication in lupus-prone mice (Lechner et al. 2000). Shanks et al. (1999) showed a decrease in hypothalamic CRH mRNA expression and an increase in arginine-vasopressin (AVP) mRNA expression associated with increased autoantibody levels, and disease progression in a SLE murine model.

*Human studies*. Zietz et al. (2000) investigated ACTH, androstenedione (ASD), cortisol, or DHEAS before and during a CRH test in patients with moderately active SLE treated with low dose of GC therapy, and their relation to IL-6 or TNF. They found marked adrenal insufficiency and a shift in steroidogenesis to cortisol in SLE patients; but a completely normal pituitary function. This finding depends in part of GC therapy.

The cortisol response after hypoglycemia was significantly lower in active SLE suggesting some degree of HPA axis dysfunction (Gutierrez et al. 1998). Recently the total anterior pituitary reserve was investigated in SLE before the initiation of GC or immunosuppressive treatment. Surprisingly no significant alteration of the HPA axis was found in SLE, which is inadequate due to continuing inflammation (Köller et al. 2004).

Straub et al. (2004) demonstrated in RA and SLE patients that low serum levels of adrenal androgens and cortisol were not due to increased renal clearance. Decreased adrenal production or increased conversion or conjugation to downstream hormones was the most likely in RA and SLE patients (Straub et al. 2004). Patients with SLE had increased neuropeptide Y levels. In contrast, plasma ACTH and cortisol levels were decreased in SLE and RA. This study displayed an increased outflow of the sympathetic nervous system and a decreased tone of the HPA axis in patients with SLE and RA (Harle et al. 2006).

Gladman et al. (1991) found that mGCR of leukocytes of SLE patients were significantly high without correlation with disease activity. However, other studies showed that mGCR was associated with active SLE (Tanaka et al. 1992). Polymorphism analysis revealed a single mutation in exon 9 of the mGR gene in SLE patients (Lee et al. 2004). Finally, Spies et al. (2006) investigated the expression of mGCR and demonstrated that these are up-regulated in SLE-patients and down-regulated by GC.

*Sjögren syndrome*

To investigate HPA axis in Sjögren syndrome (SS), the ACTH response to ovine CRH (oCRH) has been used as a direct measure of corticotrophic function, and the plasma cortisol response to the ACTH released during oCRH stimulation as an indirect measure of adrenal function. Patients with SS were characterized by significantly lower ACTH and cortisol levels, respectively. Furthermore, a blunted pituitary and adrenal response to oCRH was observed, indicating a relatively hyporesponsiveness of the adrenal gland to CRH stimulation. These findings indicate hypoactivity of the HPA axis in patients with SS (Johnson et al. 1998; Johnson et al. 2000).

In conclusion, dysfunction of HPA-axis in patients with RA, SLE and SS seems to be important in the pathogenesis of these diseases. In RA the accumulated evidence strongly supports the viewpoint that HPA-axis is deficient. Surprisingly, few studies have directly evaluated the HPA axis in SLE compared with healthy controls. Because of the small number of studies and patients, the results must be considered with caution (Li et al. 2005). The few studies available in SS patients have indicated a central deficiency in HPA axis.

### The hypothalamic pituitary gonadal axis and autoimmune rheumatic diseases

The HPG axis and sex hormones have an important immunoregulatory role. Physiological concentrations of E enhance immune responses, on the contrary, progesterone (PG) and A, such as T and DHEA, are natural immunosuppressors (Cutolo and Wilder 2000; Eskandari et al. 2003). Animal models demonstrated *in vivo* modulation of the immune system by E receptors. Knockout mouse models to E receptors α and β, indicate that both are important for thymus development and atrophy (Erlandsson et al. 2001).

*HPG and stress*

Immune stress has an inhibitory effect on the HPG axis and thus gonadal function is reduced in severe inflammation. It is mediated through a direct cytokine effect on hypothalamic neurons secreting luteinizing hormone or CRH, endogenous opioids and also by the cytokines action directly on the gonads (Rettori et al. 1991).

*Estrogens and autoimmune diseases*

Several physiological, pathological and therapeutic conditions may change the serum estrogen milieu and/or peripheral conversion rate, including the menstrual cycle, pregnancy, postpartum period, menopause, elderly, chronic stress, inflammatory cytokines, use of corticosteroids, oral contraceptives and steroid hormonal replacements, including androgen/estrogen ratios and related effects (Cutolo et al. 2004).

*Animal models*

Experimental studies try to explain the mechanisms by which E enhance the immune/inflammatory response exerted by activating the NF-kB complex pathway. Locally increased E may produce activating effects on synovial cell proliferation, including macrophages and fibroblasts (Cutolo et al. 2003). Macrophages release cytokines can be modulated by E in different ways. Fcγ receptor type IIIA (CD16a) is expressed on macrophages that selectively bind IgG molecules. Binding of CD16 by anti-CD16 monoclonal Ab stimulates macrophage cytokine release. E also modulate pro-inflammatory cytokine release from activated monocytes or macrophages through the modulation of CD16 expression (Kramer et al. 2004). There is evidence for the direct effect of E2 on B-cell function in experimental lupus, through the break of B-cell tolerance that induce a lupus-like phenotype in non-autoimmune mice (Bynoe et al. 2000). Grimaldi et al. (2001) demonstrated that E2 treatment of BALB/c mice transgenic for the heavy chain of a pathogenic anti-DNA Ab induces a lupus-like phenotype with expansion of anti-DNA B cells, elevation of anti-DNA Ab titers and glomerular immunoglobulin deposition. A sustained increase in E2 resulted in an altered distribution of B cell subsets, with a diminished transitional population and an increase in marginal zone B cells. Recent data provide evidence that E2 facilitates the maturation of a pathogenic naive autoreactive B cell repertoire and hampers the maturation of a potentially protective B cell repertoire. These data show that both, positive and negative selection occur within the transitional B cell stage under the influence of E2 in this model (Grimaldi et al. 2006).

The model of E deficiency in rodents is the aromatase-knockout (ArKO) mouse. A recent report showed that ArKO mice with E deficiency develop severe autoimmune exocrinopathy resembling SS, and suggest that E might have clinical value in the prevention or treatment of this disease (Shim et al. 2004).

*Human studies*

Clinical studies suggest a role for E in ARD. Elevated serum concentrations of 16α-hydroxyestrone have been described in patients with SLE, suggesting that abnormal patterns of E metabolism may lead to increased estrogenic activity (Lahita et al. 1979). Increased E formation and E/A ratio in the synovial fluid of RA patients have been demonstrated, probably due to increased aromatase activity (Castagnetta et al. 2003). Tengstrand et al. (2003) showed decreased DHEAS and estrone concentrations and increased E2 levels in male patients with RA. E2 correlated with indices of inflammation. In addition, RA and SLE patients have a shift to mitogenic E such as 16α-hydroxyestrone in relation to endogenous antiestrogens, which contributes to maintenance of the proliferative state in these diseases (Weidler et al. 2004). The relationship between E and E receptors (ER) may play a role in the patoghenesis of SLE. ERα and ERβ are expressed in immune cells. A recent study shows that ERα codon 594 genotype may influence the development of SLE at a younger age (Kassi et al. 2005). In primary Sjögren syndrome (pSS) the relations with E are not clear. In a study, higher levels of disease activity were associated with higher concentrations of T without correlation between E levels (Brennan et al. 2003). The apoptotic pathway may play a central role in tolerant T cells to tissue-specific self antigen, and may drive the autoimmune phenomenon. T exerted pro-apoptotic effects and reduced macrophage proliferation, whereas E2 induced anti-apoptotic effects by interfering with NF-kB activities (Cutolo et al. 2005). *In vitro* apoptosis of PBMCs in short-term unstimulated cultures of SLE cells is accelerated compared to normal individuals; however, E decrease *in vitro* apoptosis (Evans et al. 1997). Another study mentions apoptosis and E deficiency in pSS with possible antiestrogenic actions as a potent factor in the formation of pathogenic autoantigens (Hayashi et al. 2004). Two patients who received E therapy developed SS, suggesting that E may play a role in the pathogenesis of SS in some patients (Nagler and Pollack 2000).

*Androgens and autoimmune disease*

DHEAS and related steroid hormones have a variety of immunological effects both *in vitro*, experimental models, and humans (Dillon 2005).

*Animal models*

Autoimmune mice models show effects of A on immune maturation reflected by the expression of receptors for E and DHT, effects on the size and composition of the thymus, inhibition of B cells and T-cells maturation, and reduce synthesis of immunoglobulins. On monocytes, androgens decreased IL-6 and IL-1 production and on macrophages inhibit IL-1 (Ahmed et al. 1985; Grossman 1988). T specifically

Table I. Estrogen and androgen effects on immune function.

| Estrogens | Androgens |
|---|---|
| Stimulation of polyclonal B-cell activity | B-cell precursor depletion in bone marrow |
| Reduction of phytohemagglutinin response | Supress the phytohemagglutinin-induced blast transformation of lymphocytes |
| Enhanced mixed lymphocyte reaction | Thymic atrophy, thymocyte apoptosis |
| Enhances bone marrow graft rejection | Delay graft rejection in animal models |
| Enhances TH2 pathway | Enhances suppressor T cells |
| Increases the release of inflammatory cytokines | Decreases cytokine production |

targets CD8+CD4+ thymocytes for apoptosis via upgrading TNF-α production (Guevara Patino et al. 2000). Michalski et al. (1983), reported that A therapy in B/W mice is associated with improve cell-mediated immune function and increase survival. Androgen may prolong survival in this model through an effect on anomalous suppressive regulatory cells that impaired T lymphocytes function (Verthelyi and Ahmed 1994).

### *Human studies*

Human studies show low gonadal and adrenal A levels as well as reduced A/E ratio, detected in body fluids such as blood, synovial fluid, smears, saliva of male and female RA patients, supporting the possible role of gonadal hormones on the RA pathogenesis (Cutolo et al. 2002). Identification of functional A receptors in synoviocytes and the modulatory effect of DHT on the inflammatory process in the joint suggest a direct link between hypoandrogenicity and RA disease status (Khalkhali-Ellis et al. 2002). Deficiency of DHEA and DHEAS has been associated with other illnesses, therefore, some investigators have said that SLE in men are the result of too little A and too much E (Inman et al. 1982), however most studies indicate that men with SLE have normal levels of A and E/A ratios might be minimally elevated in some men; studies of A metabolism in women with SLE indicate that a difference in the overall metabolism of A can be found in this gender (Lahita 2000). Male RA patients have hypogonadism with low levels of luteinizing hormone, suggesting a central origin of the relative hypoandrogenicity (Tengstrand et al. 2002). There is controversy about the role of A in male patients with ankylosing spondylitis (AS). Gooren et al. (2000) found elevated DHEAS and 17 alpha-hydroxyprogesterone levels in AS patients and it may be secondary to inflammation and stress, or causally related to AS. However, other studies reported no alterations of serum levels of adrenal and gonadal hormones in patients with AS. The impact of sex steroids on AS is still unresolved (Straub et al. 2002). Other ARD in which low levels of DHEAS have been reported are pSS and polymyalgia rheumatica (Valtysdottir et al. 2001; Cutolo et al. 2002). In summary, sex hormones play an important role on ARD, depending on the physiological or supraphysiological concentrations, under stress conditions and other factors. The effects of estrogens and androgens are shown in Table I.

### Prolactin and autoimmune rheumatic diseases

In 1930, Smith observed in rats thymus atrophy after hypophysectomy. This was the first study to indicate the role of PRL in thymic physiology. However, until 1980s the relationship between PRL and the immune system was analysed. The classic studies of Berczi and Nagy (1982) showed that PRL is involved in the pathogenesis of AA and it restores immunocompetence in hypophysectomized or Bromocriptine (BRC) treated rats. Elevated PRL serum levels in AA, collagen type II-induced arthritis, murine and human SLE, and autoimmune type I diabetes may influence the outcome of the disease. It has been suggested that hyperprolactinemia (HPRL) is a risk factor for the development of autoimmunity and ARD (Jara et al. 1991; Neidhart 1998).

### *Animal models*

The role of PRL in murine SLE and pituitary transplantation was studied by McMurray et al. (1991). HPRL was associated with increased IgG concentrations, anti-DNA Ab, immune complex, glomerulonephritis and accelerated mortality. BRC-induced suppression of PRL was associated with decreased of disease activity, and prolonged lifespan. BRC also was beneficial in a murine model of primary antiphospholipid syndrome (Blank et al. 1995). Autoimmune disease acceleration by PRL is incremented by E stimulation (McMurray 2001). To identify whether E effects reflect increased PRL secretion, Peeva et al. (2000) showed that treatment of transgenic mice with E plus BRC led to reduced titers of anti-DNA Ab and diminished IgG deposition in kidneys compared with treatment with E alone. The E-induced breakdown in B-cell tolerance can be abrogated by BRC. On the contrary, treatment with PRL induced a lupus-like phenotype with an increased number of B cells, elevated serum anti-DNA Ab, and glomerulonephritis (Peeva et al. 2003). The spleens of treated mice displayed that marginal zone and follicular B cells produce anti-DNA Abs. Anti-DNA B cells in E-treated mice become marginal zone cells while identical cells from PRL-treated mice become follicular cells (Venkatesh et al. 2006). Taken together, these findings support the hypothesis that PRL and E favors the rupture of immune tolerance, which is a key feature of ARD.

*Human studies*

Human PRL observations in ARD, particularly in SLE, preceded to murine SLE studies. The first report of HPRL in SLE was in men (Lavalle et al. 1987). This finding was confirmed by other studies. HPRL has been found in 20–30% of patients with SLE particularly in those patients with active diseases (Jara et al. 1992; Walker and Jacobson 2000). There is controversy if HPRL is associated with active SLE (Buskila et al. 1996; Jimena et al. 1998). This discrepant finding may be explained by different factors such as: statistical power of these studies (Blanco-Favela et al. 1999), heterogeneous groups of patients, variability of the SLE activity indices used, different treatments, abnormal circadian rhythms of PRL, anti-PRL Ab, etc. (Jara et al. 2001). SLE patients with anti-PRL Ab could be the cause of HPRL in a subset of SLE patients (Leanos et al. 1998). New evidence has confirmed a significant correlation between bioactive and immunoreactive PRL levels and SLE activity (Jacobi et al. 2001; Leanos-Miranda et al. 2001; Pacilio et al. 2001).

Regardless of this controversy, human studies suggest that PRL can participate in major and minor organ involvement such as lupus nephritis, neuropsychiatry lupus, joint and cutaneous lupus (Jara et al. 1998; Miranda et al. 1998; Vera-Lastra et al. 2003). Similar observations have been described in synovium infiltrating T cells in RA (Nagafuchi et al. 1999). These clinical observations suggest that PRL may be produced in organ affected from SLE and others ARD, by infiltrating lymphocytes.

In this regard, PBMCs from patients with active SLE have increased production of PRL-like immunoreactive substance with different molecular weight: 11, 60 kDa and it seems to be derived from B lymphocytes (Gutierrez et al. 1995; Larrea et al. 1997). PRL with 130 and 23 kDa were associated with inactive SLE (Cruz et al. 2001). An increase in serum free PRL levels, with high levels of little PRL and low levels of big/big PRL was associated with lupus activity (Leanos-Miranda and Cardenas-Mondragon 2006). In contrast, other study showed that patients with increased big/big form had a very active illness (Garcia et al. 2004).

The exact origin of HPRL in SLE patients is unknown and these studies suggest that active SLE lymphocytes may be the source of PRL. In support of this hypothesis, we found a significant decreased of PRL concentrations following treatment with immunosuppressive drugs (Vera-Lastra et al. 2003), SLE lymphocytes synthesize PRL and have alterations in the CREB family proteins, which participate in lymphocyte PRL gene expression (Gutierrez et al. 1995; Larrea et al. 1997; Montgomery 2001) and a functionally significant polymorphism is over expressed in SLE lymphocytes (Stevens et al. 2001). Lymphocyte-derived PRL might contribute to alter the functional activity of the hypothalamic dopaminergic system in SLE attempting to maintain serum PRL within a physiological range (Mendez et al. 2004). These evidences provide a potential explanation for the increased PRL release by SLE lymphocytes. However, is not known if lymphocytes can produce enough PRL to cause an increase in serum PRL levels. In this regard, it has been demonstrated HPRL and PRL-immunostaining in bone marrow cells of patients with multiple myeloma. PRL-immunopositive bone marrow cells disappeared and serum PRL normalized after treatment (Gado et al. 2001). This study suggests that B lymphocytes are the source of HPRL in multiple myeloma.

HPRL is associated with several Ab involved in SLE such as ANAs, anti-dsDNA, anticardiolipin Ab, etc. and hypocomplementemia. PRL induces immunoglobulin synthesis and anti-dsDNA by SLE lymphocytes. Of interest, the physiological concentrations of PRL (20 ng/ml) induced IgG production more effective than PRL at 100 ng/ml (Vera-Lastra et al. 2002). HPRL in SLE and RA is associated with antithyroid Ab, evidencing the association of PRL and autoimmunity (Kramer et al. 2005).

The role of PRL in the lymphocytes activation in active and inactive SLE was studied in an *in vitro* model. CD69 expression was associated with disease activity. In contrast, CD154 did not show this association. Mononuclear cells were capable of producing and secreting PRL and to express PRL-receptor (Chavez-Rueda et al. 2005).

Of interest, the PRL gene is in close proximity to the HLA region on the short arm of chromosome 6. Linkage disequilibrium between HLA–DRB1 (alleles associated with SLE and RA susceptibility) and PRL genes (Brennan et al. 1997) has been described. This report provides an immunogenetic base to explain the relationship between SLE and PRL. However, a recent analysis of PRL and PRL-receptor genes polymorphism in SLE and multiple sclerosis did not find statistically significant difference in the allele distribution (Mellai et al. 2003).

HPRL was found in patients with RA, reactive arthritis, systemic sclerosis (SSc), Sjogren syndrome and juvenile chronic arthritis (Walker and Jacobson 2000). Patients with AS had significant lower serum levels of osteocalcin and higher levels of serum T, LH and PRL (El Maghraoui et al. 2005). Recently, we analysed the status of the hypothalamic dopaminergic tone using metoclopramide (MTC) test and the prevalence of pituitary adenomas in SSc. A group of patients with SSc have a high prevalence of HPRL with increased central dopaminergic tone, and microadenomas (Vera-Lastra et al. 2006). In this regard, microadenomas have been found in patients with SLE and other rheumatic diseases. Patients with long-standing HPRL secondary to microadenoma may develop SLE. In some patients, SLE onset was preceded by withdrawal or tapering BRC (Walker and Jacobson 2000; Jara et al. 2001). The first pediatric case of prolactinoma associated with SLE, in a 13-year-old white female was reported. Neurosurgical resection and medical therapy with BRC were independently associated with decreased PRL levels and remission of clinical and serological SLE manifestations (Reuman 2004). Three other patients with SLE and prolactinomas have been reported without findings related to HPRL or its association with SLE flares. This may be due to genetic differences in the response to PRL and/or to the presence of variant PRL isoforms that have reduced biological activity (Li et al. 2006). Finally, a patient with hypocomplementemic urticarial vasculitis, Jaccoud's arthropathy, SLE and Sjogren syndrome in association with HPRL secondary to pituitary microadenoma has been described. The genetic studies of this patient disclosed HLA class II DRB1* 0301 QDB1* 0201. The combination of genetic susceptibility, female gender, multiple autoimmune disease and HPRL represent a classic example of the concept of autoimmunity developing as a mosaic (Anaya and Shoenfeld 2005).

In conclusion, *in vitro* experimental models and clinical evidences strongly support the role of PRL in the pathogenesis and clinical manifestations of SLE and other ARD.

### Immune neuroendocrine system, pregnancy and autoimmune rheumatic diseases

Endocrine regulation of fetal growth involves interactions between the mother, placenta and fetus, and these effects may program long-term physiology (Murphy et al. 2006). Many hormones are increased during pregnancy such as CRH, pro-opiomelanocortin, ACTH, cortisol, E, PG, PRL and human placental lactogen. The increases of E, PG and GC during pregnancy are associated with increased production of Th2 cytokines, and decreased production of Th1 cytokines. These patterns reverse in the postpartum period (Kanik and Wilder 2000). Evidence acquired from clinical data and experimental animal models suggest that Th1 cytokines are detrimental to pregnancy while Th2 cytokines, are protective (Wegmann et al. 1993). Detection of IL-15 and IL-18 at the placental level suggest that interactions between cytokines and hormones is more complex that the Th1/Th2 paradigm to explain the pregnancy course. IL-15 has been implicated in differentiation and proliferation of uterine NK cells, while IL-18 enhanced innate immunity (Laskarin et al. 2005). The effects of cytokines on reproductive events led to the "immunotrophism" model, which suggests that maternal immune recognition of fetal-derived Ags results in the release of cytokines that promote the growth of the placenta (Tangri et al. 1994). During pregnancy there is an up regulation of PG receptors on activated lymphocytes among placental cells and decidual CD56+ cells. In presence of sufficient PG, these cells synthesize progesterone induced blocking factor (PIBF), a mediator that exerts substantial anti-abortive activities. PIBF affects B cells and induces an increased production of non-cytotoxic Ab and it also alters the profile of cytokine secretion by activated lymphocytes resulting in an increase in the production of non-inflammatory, non-cytotoxic cytokines and a reduction in the production of inflammatory, cytotoxic cytokines (Druckmann and Druckmann 2005).

Only in pregnancy experimental models, there are detailed studies regarding the responsiveness to the stressors of the HPA axis. In late pregnant rats and during lactation, HPA axis is hypo responsive to psychogenic stressors (Neumann et al. 1998) and the responses to immune signals (LPS, IL-1) are suppressed. *In situ* hybridization revealed decreased CRH and pro-opiomelanocortin mRNA expression in the hypothalamus. In contrast, proenkephalin-A and mu-opioid receptor mRNA expression in the nucleus tractus solitarius (NTS) was significantly increased in pregnant rats, indicating that the HPA axis responses to immune signals are suppressed in pregnancy at the level of hypothalamic CRH neurons through an opioid mechanism (Brunton et al. 2005). Additionally, activation of neuropeptidergic systems such as oxytocin and PRL, which are necessary for reproductive processes (labor, lactogenesis, maternal behavior), may be involved in the inhibition of ACTH and GC secretory responses (Neumann et al. 2003). These adaptations preserve the oxytocin store for parturition and prevent pre-term birth (Russell and Brunton 2006). PRL has recently been shown to exert an anxiolytic effect and an inhibitory tone on HPA axis activity in pregnancy and lactation with increased expression of the hypothalamic PRL gene, suggesting an activated state of PRL during the peripartum period. Activation of the PRL in the peripartum period significantly contributes to emotional and neuroendocrine adaptations, with down regulation of the responsiveness of the HPA axis (Torner et al. 2002).

In 1991, we demonstrated that gonadal hormones and PRL alterations during pregnancy are present in SLE patients. These changes may be related to fetal wastage and disease activity. Significant lower serum E2 and testosterone with higher PRL levels in pregnant patients with SLE were found (Jara-Quezada et al. 1991). A longitudinal analysis of PRL in SLE pregnancy confirmed the association of PRL with pregnant lupus disease activity (Petri and Chan 1995). Doria et al. (2002), confirmed the variation of steroid hormone levels during pregnancy in patients with SLE. Disease activity score significantly varied during pregnancy and postpartum In SLE patients, E2, PG and DHEAS concentrations were found to be significantly reduced. This finding probably was due to placental insufficiency. These steroid hormone variations may result in a lower humoral immune response activation, which in turn could account for the decrease in disease activity observed during the third trimester in pregnant SLE patients.

McMurray et al. (1993) developed elegant animal experiments. Pregnancy, with or without suckling and pseudopregnancy induced long-term HPRL, which accelerate the appearance of anti-DNA Ab to the viral protein gp70, hypergammaglobulinemia and disease activity. Taken together, clinical and experimental results leave little doubt that in pregnancy SLE, PRL indeed contributes to the pathogenesis of disease (Berczi 1993).

A reduction in the incidence and severity of RA is seen in pregnant women. Relaxin, a hormone of pregnancy, has been implicated in decreased immune responsiveness. Relaxin and estradiol valerate therapy reduced AA (Santora et al. 2005). In a prospective study in RA, SLE and normal pregnant women, TNFα was found in all groups studied. High IL-10 levels were found in RA and SLE patients. This study showed decreased cortisol levels in all patients. Immune and hormonal networks are involved in pregnancy and ARD and these are very dynamic immune processes (Munoz-Valle et al. 2003).

The risk of RA seems to be associated with reduced fecundity and with breastfeeding; these apparently contradictory risk factors can be explained by their association with high PRL concentrations. The effects of breastfeeding and nulliparity are modified by HLA DR4 status, suggesting an interaction between genetic and reproductive risk factors in the etiology of RA. The associations between DR4 and reproductive risk factors in RA are due to linkage disequilibrium between DR4 and an abnormally regulated PRL gene polymorphism (Brennan et al. 1996).

During pregnancy, experimental models and patients with ARD develop an abnormal immune-neuroendocrine response. In SLE, the contribution of E, PG, A and PRL may influence to successful or pathologic pregnancy. However a multitude of questions remain to be investigated (McMurray 2002).

### Conclusion and perspectives

Studies accumulated from two decades ago and during the last years support a role of immune-neuroendocrine system in the pathogenesis and clinical expression of ARD. During inflammatory stimuli, the interaction among HPA, HPG, HPT and PRL-gonadal axis with immune system is abnormal in patients with ARD, particularly during active disease. Table II shows the alterations of neuroendocrine system in ARD. The ARD are caused by a failure of immune cells tolerance, and the abnormal response of immune-neuroendocrine system may participate in the break of tolerance. These findings have implications for the treatment of autoimmune disease.

Table II. Neuroendocrine alterations in ARD.

| Disease | HPA axis | HPG axis | PRL axis |
|---|---|---|---|
| SLE | ↓GC, ↓ACTH, ↓CRH | ↑ E, ↓ A | ↑ PRL |
| RA | ↓GC, ↓ACTH ↓ CRH, | ↓ E ↓ A | ↑ ↓PRL |
| SS | ↓GC, ↓ ACTH | ↓ E ↑ A | ↑ PRL |
| AS | | ↑ PG, ↑ A | ↑ PRL |

### References

Ahmed SA, Dauphinee M, Talal N. 1985. Effects of short term administration of sex hormones on normal and autoimmune mice. J Immunol 134:204–210.

Anaya JM, Shoenfeld Y. 2005. Multiple autoimmune disease in a patient with hyperprolactinemia. Isr Med Assoc J 7:740–741.

Bartholome B, Spies CM, Gaber T, Schuchmann S, Berki T, Kunkel D, et al. 2004. Membrane glucocorticoid receptors (mGCR) are expressed in normal human peripheral blood mononuclear cells and up-regulated after *in vitro* stimulation and in patients with rheumatoid arthritis. FASEB J 18:70–80.

Berczi I, Nagy E. 1982. A possible role of prolactin in adjuvant arthritis. Arthritis Rheum 25:591–594.

Berczi I. 1993. Prolactin, pregnancy and autoimmune disease. J Rheumatol 20:1095–1100.

Besedovsky HO, del Rey A. 1996. Immune-neuro-endocrine interactions: Facts and hypotheses. Endocr Rev 17:64–102.

Blanco-Favela F, Quintal-Alvarez G, Leanos-Miranda A. 1999. Association between prolactin and disease activity in systemic lupus erythematosus. Influence of statistical power. J Rheumatol 26:55–59.

Blank M, Krause I, Buskila D, Teitelbaum D, Kopolovic J, Afek A, et al. 1995. Bromocriptine immunomodulation of experimental SLE and primary antiphospholipid syndrome via induction of nonspecific T suppressor cells. Cell Immunol 162:114–122.

Brennan P, Ollier B, Worthington J, Hajeer A, Silman A. 1996. Are both genetic and reproductive associations with rheumatoid arthritis linked to prolactin? Lancet 348:106–109.

Brennan P, Hajeer H, Ong KR, et al. 1997. Allelic markers close to prolactin are associated with HLA-DRB1 susceptibility alleles among women with rheumatoid arthritis and systemic lupus erythematosus. Arthritis Rheum 40:1383–1386.

Brennan MT, Sankar V, Leakan RA, Grisius MM, Collins MT, Fox PC, Baum BJ, Pillemer SR. 2003. Sex steroid hormones in primary Sjogren's syndrome. J Rheumatol 30:1267–1271.

Brunton PJ, Meddle SL, Ma S, Ochedalski T, Douglas AJ, Russell JA. 2005. Endogenous opioids and attenuated hypothalamic-pituitary-adrenal axis responses to immune challenge in pregnant rats. J Neurosci 25:5117–5126.

Buskila D, Lorber M, Neumann L, Flusser D, Shoenfeld Y. 1996. No correlation between prolactin levels and clinical activity in patients with systemic lupus erythematosus. J Rheumatol 23:629–632.

Bynoe MS, Grimaldi CM, Diamond B. 2000. Estrogen up-regulates Bcl-2 and blocks tolerance induction of naïve B cells. Proc Natl Acad Sci USA 97:2703–2708.

Castagnetta LA, Carruba G, Granata OM, Stefano R, Miele M, Schmidt M, et al. 2003. Increased estrogen formation and estrogen to androgen ratio in the synovial fluid of patients with rheumatoid arthritis. J Rheumatol 30:2597–2605.

Chavez-Rueda K, Hernandez J, Zenteno E, Leanos-Miranda A, Legorreta-Haquet MV, Blanco-Favela F. 2005. Identification of prolactin as a novel immunomodulator on the expression of co-stimulatory molecules and cytokine secretions on T and B human lymphocytes. Clin Immunol 116:182–191.

Chavez-Rueda K, Legorreta-Haquet MV, Cervera-Castillo H, Sanchez L, Jara LJ, Zenteno E, et al. 2005. Prolactine effect on CD69 and CD154 expression by CD4+ cells from systemic lupus erythematosus patients. Clin Exp Rheumatol 23: 769–777.

Chida D, Imaki T, Suda T, Iwakura Y. 2005. Involvement of corticotropin-releasing hormone- and interleukin (IL)-6-dependent proopiomelanocortin induction in the anterior pituitary during hypothalamic-pituitary-adrenal axis activation by IL-1alpha. Endocrinology 146:5496–5502.

Chikanza IC, Grossman AB. 2000. Reciprocal interactions between the neuroendocrine and immune systems during inflammation. Rheum Dis Clin North Am 26:693–711.

Chikanza IC, Petrou P, Kingsley G, Chrousos GP, Panayi GS. 1992. Defective hypothalamic response to immune and inflammatory stimuli in patients with rheumatoid arthritis. Arthritis Rheum 35:1281–1288.

Chover-Gonzalez AJ, Jessop DS, Tejedor-Real P, Gibert-Rahola J, Harbuz MS. 2000. Onset and severity of inflammation in rats exposed to the learned helplessness paradigm. Rheumatology (Oxford) 39:764–771.

Chrousos GP, Kino T. 2005. Ikaros transcription factors: Flying between stress and inflammation. J Clin Invest 115:844–848.

Cremaschi GA, Gorelik G, Klecha AJ, Lysionek AE, Genaro AM. 2000. Chronic stress influences the immune system through the thyroid axis. Life Sci 67:3171–3179.

Cruz J, Avina-Zubieta A, Martinez de la Escalera G, Clapp C, Lavalle C. 2001. Molecular heterogeneity of prolactin in the plasma of patients with systemic lupus erythematosus. Arthritis Rheum 44:1331–1335.

Cutolo M, Wilder RL. 2000. Different roles for androgens and estrogens in the susceptibility to autoimmune rheumatic diseases. Rheum Dis Clin North Am 26:825–839.

Cutolo M, Seriolo B, Villaggio B, Pizzorni C, Craviotto C, Sulli A. 2002. Androgens and estrogens modulate the immune and inflammatory responses in rheumatoid arthritis. Ann NY Acad Sci 966:131.

Cutolo M, Foppiani L, Minuto F. 2002. Hypothalamic-pituitary-adrenal axis impairment in the pathogenesis of rheumatoid arthritis and polymyalgia rheumatica. J Endocrinol Invest 25(10 Suppl):19–23.

Cutolo M, Capellino S, Montagna P, Villaggio B, Sulli A, Seriolo B, et al. 2003. New roles for estrogens in rheumatoid arthritis. Clin Exp Rheumatol 21:687–690.

Cutolo M, Sulli A, Capellino S, Villaggio B, Montagna P, Seriolo B, et al. 2004. Sex hormones influence on the immune system: Basic and clinical aspects in autoimmunity. Lupus 13:635–638.

Cutolo M, Capellino S, Montagna P, Ghiorzo P, Sulli A, Villaggio B. 2005. Sex hormone modulation of cell growth and apoptosis of the human monocytic/macrophage cell line. Arthritis Res Ther 7:R1124–R1132.

De Bellis A, Bizzarro A, Pivonello R, Lombardi G, Bellastella A. 2005. Prolactin and autoimmunity. Pituitary 8:25–30.

Dillon JS. 2005. Dehydroepiandrosterone, dehydroepiandrosterone sulfate and related steroids: Their role in inflammatory, allergic and immunological disorders. Curr Drug Targets Inflamm Allergy 4:377–385.

Doria A, Cutolo M, Ghirardello A, Zampieri S, Vescovi F, Sulli A. 2002. Steroid hormones and disease activity during pregnancy in systemic lupus erythematosus. Arthritis Rheum 47:202–209. Apr 15.

Druckmann R, Druckmann MA. 2005. Progesterone and the immunology of pregnancy. J Steroid Biochem Mol Biol 97:389–396.

Eijsbouts AM, van den Hoogen FH, Laan RF, Hermus AR, Seep CG, van de Putte LB. 2005. Hypothalamic-pituitary-adrenal axis activity in patients with rheumatoid arthritis. Clin Exp Rheumatol 23:658–664.

El Maghraoui A, Tellal S, Chaouir S, Lebbar K, Bezza A, Nouijai A, et al. 2005. Bone turnover markers, anterior pituitary and gonadal hormones, and bone mass evaluation using quantitative computed tomography in ankylosing spondylitis. Clin Rheumatol 24:346–351.

Elenkov IJ, Iezzoni DG, Daly A, Harris AG, Chrousos GP. 2005. Cytokine dysregulation, inflammation and well-being. Neuroimmunomodulation 12:255–269.

Erlandsson MC, Ohlsson C, Gustafsson JA, Carlsten H. 2001. Role of oestrogen receptors alpha and beta in immune organ development and in oestrogen-mediated effects on thymus. Immunology 103:17–25.

Eskandari F, Webster JI, Sternberg EM. 2003. Neural immune pathways and their connection to inflammatory diseases. Arthritis Res Ther 5:251–265.

Evans MJ, MacLaughlin S, Marvin RD, Abdou NI. 1997. Estrogen decreases *in vitro* apoptosis of peripheral blood mononuclear cells from women with normal menstrual cycles and decreases TNF-alpha production in SLE but not in normal cultures. Clin Immunol Immunopathol 82:258–262.

Ezzat S, et al. 2005. Ikaros integrates endocrine and immune system development. J Clin Invest 115:1021–1029, doi:10.1172/JCI200522486.

Francis K, Lewis BM, Akatsu H, Monk PN, Cain SA, Scanlon MF, Morgan BP, Ham J, Gasque P. 2003. Complement C3a receptors in the pituitary gland: A novel pathway by which an innate immune molecule releases hormones involved in the control of inflammation. FASEB J 17:2266–2268.

Gado K, Rimanoczi E, Hasitz A, Gigler G, Toth BE, Nagy GM, Paloczi K, Domjan G. 2001. Elevated levels of serum prolactin in patients with advanced multiple myeloma. Neuroimmunomodulation 9:231–236.

Garcia M, Colombani-Vidal ME, Zylbersztein CC, Testi A, Marcos J, Arturi A, Babini J, Scaglia HE. 2004. Analysis of molecular heterogeneity of prolactin in human systemic lupus erythematosus. Lupus 13:575–583.

Gerlo S, Verdood P, Hooghe-Peters EL, Kooijman R. 2005. Modulation of prolactin expression in human T lymphocytes by cytokines. J Neuroimmunol 162:190–193.

Gladman DD, Urowitz MB, Doris F, Lewandowski K, Anhorn K. 1991. Glucocorticoid receptors in systemic lupus erythematosus. J Rheumatol 18:681–684.

Gooren LJ, Giltay EJ, van Schaardenburg D, Dijkmans BA. 2000. Gonadal and adrenal sex steroids in ankylosing spondylitis. Rheum Dis Clin North Am 26:969–987.

Grimaldi CM, Michael DJ, Diamond B. 2001. Cutting edge: Expansion and activation of a population of autoreactive marginal zone B cells in a model of estrogen-induced lupus. J Immunol 167:1886–1890.

Grimaldi CM, Jeganathan V, Diamond B. 2006. Hormonal regulation of B cell development: 17beta-estradiol impairs negative selection of high-affinity DNA-reactive B cells at more than one developmental checkpoint. J Immunol 176: 2703–2710.

Grossman CJ. 1988. The importance of hormones in the regulation of the immune system. Immunol Allergy Pract 10:104–106.

Guevara Patino JA, Marino MW, Ivanov VN, Nikolich-Zugich J. 2000. Sex steroids induce apoptosis of CD8+CD4+

double-positive thymocytes via TNF-alpha. Eur J Immunol 30: 2586–2592.

Gutierrez MA, Molina JF, Jara LJ, Cuellar ML, Garcia C, Gutierrez-Urena S, Gharavi A, Espinoza LR. 1995. Prolactin and systemic lupus erythematosus: Prolactin secretion by SLE lymphocytes and proliferative (autocrine) activity. Lupus 4: 348–352.

Gutierrez MA, Garcia ME, Rodriguez JA, Rivero S, Jacobelly S. 1998. Hypothalamic-pituitary-adrenal axis function and prolactin secretion in systemic lupus erythematosus. Lupus 7: 404–408.

Gutierrez MA, Garcia ME, Rodriguez JA, Mardonez G, Jacobelli S, Rivero S. 1999. Hypothalamic-pituitary-adrenal axis function in patients with active rheumatoid arthritis: A controlled study using insulin hypoglycemia stress test and prolactin stimulation. J Rheumatol 26:277–281.

Hansson GK, Libby P, Schonbeck U, Yan ZQ. 2002. Innate and adaptive immunity in the pathogenesis of atherosclerosis. Circ Res 91:281–291.

Harle P, Straub RH, Wiest R, Maver A, Scholmerich J, Atzeni F, et al. 2006. Increase of sympathetic outflow measured by neuropeptide Y and decrease of the hypothalamic-pituitary-adrenal axis tone in patients with systemic lupus erythematosus and rheumatoid arthritis: Another example of uncoupling of response systems. Ann Rheum Dis 65:51–56.

Hayashi Y, Arakaki R, Ishimaru N. 2004. Apoptosis and estrogen deficiency in primary Sjögren syndrome. Curr Opin Rheumatol 16:522–526.

Hoebe K, Janssen E, Beutler B. 2004. The interface between innate and adaptive immunity. Nat Immunol 5:971–974.

Inman RD, Jovanovic L, Markenson JA. 1982. Systemic lupus erythematosus in men: Genetic and endocrine features. Arch Intern Med 142:1813–1815.

Jacobi AM, Rohde W, Ventz M, Riemekasten G, Burmester GR, Hiepe F. 2001. Enhanced serum prolactin (PRL) in patients with systemic lupus erythematosus: PRL levels are related to the disease activity. Lupus 10:554–561.

Jara LJ, Lavalle C, Fraga A, Gomez-Sanchez C, Silveira LH, Martinez-Osuna P, et al. 1991. Prolactin, immunoregulation, and autoimmune diseases. Semin Arthritis Rheum 20:273–284.

Jara LJ, Gomez-Sanchez C, Silveira LH, Martinez-Osuna P, Vasey FB, Espinoza LR. 1992. Hyperprolactinemia in systemic lupus erythematosus: Association with disease activity. Am J Med Sci 303:222–226.

Jara LJ, Irigoyen L, Ortiz MJ, Zazueta B, Bravo G, Espinoza LR. 1998. Prolactin and interleukin-6 in neuropsychiatric lupus erythematosus. Clin Rheumatol 17(2):110–114.

Jara LJ, Vera-Lastra O, Miranda JM, Alcala M, Alvarez-Nemegyei J. 2001. Prolactin in human systemic lupus erythematosus. Lupus 10:748–756.

Jara-Quezada L, Graef A, Lavalle C. 1991. Prolactin and gonadal hormones during pregnancy in systemic lupus erythematosus. J Rheumatol 18:349–353.

Jimena P, Aguirre MA, Lopez-Curbelo A, de Andres M, Garcia-Courtay C, Cuadrado MJ. 1998. Prolactin levels in patients with systemic lupus erythematosus: A case controlled study. Lupus 7:383–386.

Johnson EO, Vlachoyiannopoulos PG, Skopouli FN, Tzioufas AG, Moutsopoulos HM. 1998. Hypofunction of the stress axis in Sjögren's syndrome. J Rheumatol 25:1508–1514.

Johnson EO, Skopouli FN, Moutsopoulos HM. 2000. Neuroendocrine manifestation in Sjögren syndrome. Rheum Dis Clin N Am 26:927–949.

Kanik KS, Wilder RL. 2000. Hormonal alterations in rheumatoid arthritis, including the effects of pregnancy. Rheum Dis Clin North Am 26:805–823.

Kassi E, Vlachoyiannopoulos PG, Kominakis A, Kiaris H, Moutsopulos HM, Moutsatsou P. 2005. Estrogen receptors alpha gene polymorphism and systemic lupus erythematosus: A possible risk? Lupus 14:391–398.

Khalkhali-Ellis Z, Handa RJ, Price RH Jr, Adams BD, Callaghan JJ, Hendrix MJ. 2002. Androgen receptors in human synoviocytes and androgen regulation of interleukin 1 beta (IL-1beta) induced IL-6 production: A link between hypoandrogenicity and rheumatoid arthritis? J Rheumatol 29:1843–1846.

Klecha AJ, Genaro AM, Lysionek AE, Caro RA, Coluccia AG, Cremaschi GA. 2000. Experimental evidence pointing to the bidirectional interaction between the immune system and the thyroid axis. Int J Immunopharmacol 22:491–500.

Kramer PR, Kramer SF, Guan G. 2004. 17 beta-estradiol regulates cytokine release through modulation of CD16 expression in monocytes and monocyte-derived macrophages. Arthritis Rheum 50:1967–1975.

Kramer CK, Tourinho TF, de Castro WP, da Costa Oliveira M. 2005. Association between systemic lupus erythematosus, rheumatoid arthritis, hyperprolactinemia and thyroid autoantibodies. Arch Med Res 36:54–58.

Köller MD, Templ E, Riedl M, Clodi M, Wagner O, Smolen JS, et al. 2004. Pituitary function in patients with newly diagnosis untreated systemic lupus erythematosus. Ann Rheum Dis 63:1677–1680.

Lahat N, Miller A, Shtiller R, Touby E. 1993. Differential effects of prolactin upon activation and differentiation of human B lymphocytes. J Neuroimmunol 47:35–40.

Lahita RG, Bradlow HL, Kunkel HG, Fishman J. 1979. Alterations of estrogen metabolism in systemic lupus erythematosus. Arthritis Rheum 22:1195–1198.

Lahita RG. 2000. Sex hormones and systemic lupus erythematosus. Rheum Dis Clin North Am 26:951–969.

Lang TJ. 2004. Estrogen as an immunomodulator. Clin Immunol 113:224–230.

Larrea F, Martinez-Castillo A, Cabrera V, Alcocer-Varela J, Queipo G, Carino C, et al. 1997. A bioactive 60-kilodalton prolactin species is preferentially secreted in cultures of mitogen-stimulated and nonstimulated peripheral blood mononuclear cells from subjects with systemic lupus erythematosus. J Clin Endocrinol Metab 82:3664–3669.

Laskarin G, Strbo N, Bogovic Crncic T, Juretic K, Ledee Bataille N, Chaouat G, et al. 2005. Physiological role of IL-15 and IL-18 at the maternal-fetal interface. Chem Immunol Allergy 89:10–25.

Lavalle C, Loyo E, Paniagua R, Bermudez JA, Herrera J, Graef A, et al. 1987. Correlation study between prolactin and androgens in male patients with systemic lupus erythematosus. J Rheumatol 14:268–272.

Leanos A, Pascoe D, Fraga A, Blanco-Favela F. 1998. Anti-prolactin autoantibodies in systemic lupus erythematosus patients with associated hyperprolactinemia. Lupus 7:398–403.

Leanos-Miranda A, Cardenas-Mondragon G. 2006. Serum free prolactin concentrations in patients with systemic lupus erythematosus are associated with lupus activity. Rheumatology (Oxford) 45:97–101.

Leanos-Miranda A, Pascoe-Lira D, Chavez-Rueda KA, Blanco-Favela F. 2001. Antiprolactin autoantibodies in systemic lupus erythematosus: Frequency and correlation with prolactinemia and disease activity. J Rheumatol 28:1546–1553.

Lechner O, Hu Y, Jafarian-Tehrani M, Dietrich H, Schwarz S, Herold M, et al. 1996. Disturbed immuno-endocrine communication via the hypothalamo-pituitary-adrenal axis in murine lupus. Brain Behav Immun 10:337–359.

Lechner O, Dietrich H, Oliveira dos Santos A, Wiegers GJ, Schwarz S, Harbutz M, et al. 2000. Altered circadian rhythms of the stress hormone and melatonin response in lupus-prone MRL/MP-fas(Lpr) mice. J Autoimmun 14:325–333.

Lee YM, Fujiwara J, Munakata Y, Ishii T, Sugawara A, Kaku M, et al. 2004. A mutation of the glucocorticoid receptor gene in patients with systemic lupus erythematosus. Tohoku J Exp Med 203:69–76.

Li J, May W, McMurray RW. 2005. Pituitary hormones and systemic lupus erythematosus. Arthritis Rheum 52:3701–3712.

Li M, Keiser HD, Peeva E. 2006. Prolactinoma and systemic lupus erythematosus: Do serum prolactin levels matter? Clin Rheumatol :1–4[Epub ahead of print].

Masi AT, Aldag JC, Chatterton RT, Adams RF, Kitabchi AE. 2000. Adrenal androgen and glucocorticoid dissociation in premenopausal rheumatoid arthritis: A significant correlate or precursor to onset? Z Rheumatol 59(2):II/54–II/61.

Masi AT, Aldag JC, Jacobs JWG. 2005. Rheumatoid arthritis: Neuroendocrine immune integrated physiopathogenetic. Perspectives and therapy. Rheum Dis Clin N Am 31:131–160.

Matalka KZ. 2003. Prolactin enhances production of interferon-$\gamma$, interleukin-12, and interleukin-10, but not of tumor necrosis factor-$\alpha$ in a stimulus-specific manner. Cytokine 21:187–194.

Matera L, Contarini M, Bellone G, Forno B, Biglino A. 1999. Upmodulation of interferon-$\gamma$ mediates the enhancement of spontaneous cytotoxicity in prolactin-activated natural killer cells. Immunology 98:386–392.

McMurray R, Keisler D, Kanuckel K, Izui S, Walker SE. 1991. Prolactin influences autoimmune disease activity in the female B/W mouse. J Immunol 147:3780–3787.

McMurray RW. 2001. Prolactin in murine systemic lupus erythematosus. Lupus 10:742–747.

McMurray RW, Keisler D, Izui S, Walker SE. 1993. Effects of parturition, suckling and pseudopregnancy on variables of disease activity in the B/W mouse model of systemic lupus erythematosus. J Rheumatol 20:1143–1151.

McMurray RW. 2002. Steroid hormones in lupus pregnancy: In control? Arthritis Rheum 47:116–117.

Medzhitov R, Janeway C Jr. 2000. Innate immunity. N Engl J Med 343:338–344.

Mellai M, Giordano M, D'Alfonso S, Marchini M, Scorza R, Giovanna Danieli M, et al. 2003. Prolactin and prolactin receptor gene polymorphisms in multiple sclerosis and systemic lupus erythematosus. Hum Immunol 64:274–284.

Mendez I, Alcocer-Varela J, Parra A, Lava-Zavala A, de la Cruz DA, Alarcon-Segovia D, Larrea F. 2004. Neuroendocrine dopaminergic regulation of prolactin release in systemic lupus erythematosus: A possible role of lymphocyte-derived prolactin. Lupus 13:45–53.

Michalski JP, McCombs CC, Roubinian JR, Talal N. 1983. Effect of androgen therapy on survival and suppressor cell activity in aged NZB/NZW F1 hybrid mice. Clin Exp Immunol 52:229–233.

Miranda JM, Prieto RE, Paniagua R, Garcia G, Amato D, Barile L, Jara LJ. 1998. Clinical significance of serum and urine prolactin levels in lupus glomerulonephritis. Lupus 7:387–391.

Montgomery DW. 2001. Prolactin production by immune cells. Lupus 10:665–675.

Morales P, Carretero MV, Geronimo H, Copin SG, Gaspar ML, Marcos MA, Martin-Perez J. 1999. Influence of prolactin on the differentiation of mouse B-lymphoid precursors. Cell Growth Differ 10:583–590.

Morand EF, Leech M. 2001. Hypothalamic-pituitary-adrenal axis regulation of inflammation in rheumatoid arthritis. Immunol Cell Biol 79:395–399.

Munoz-Valle JF, Vazquez-Del Mercado M, Garcia-Iglesias T, Orozco-Barocio G, Bernard-Medina G, Martinez-Bonilla G, et al. 2003. T(H)1/T(H)2 cytokine profile, metalloprotease-9 activity and hormonal status in pregnant rheumatoid arthritis and systemic lupus erythematosus patients. Clin Exp Immunol 131:377–384.

Murphy VE, Smith R, Giles WB, Clifton VL. 2006. Endocrine regulation of human fetal growth: The role of the mother, placenta, and fetus. Endocr Rev Mar 23; [Epub ahead of print].

Nagafuchi H, Suzuki N, Kaneko A, Asai T, Sakane T. 1999. Prolactin locally produced by synovium infiltrating T lymphocytes induces excessive synovial cell functions in patients with rheumatoid artritis. J Rheumatol 26:1890–1900.

Nagler RM, Pollack S. 2000. Sjögren's syndrome induced by estrogen therapy. Semin Arthritis Rheum 30:209–214.

Neeck G, Federlin K, Graef V, Rusch D, Schmidt KL. 1990. Adrenal secretion of cortisol in patients with rheumatoid arthritis. J Rheumatology 17:24–29.

Neeck G, Kluter A, Dotzlaw H, Eggert M. 2002. Involvement of the glucocorticoid receptor in the pathogenesis of rheumatoid artritis. Ann N Y Acad Sci 966:491–495.

Neidhart M. 1998. Prolactin in autoimmune diseases. Proc Soc Exp Biol Med 217:408–419.

Neumann ID, Johnstone HA, Hatzinger M, Liebsch G, Shipston M, Russell JA, et al. 1998. Attenuated neuroendocrine responses to emotional and physical stressors in pregnant rats involve adenohypophysial changes. J Physiol 508(Pt 1):289–300.

Neumann ID, Bosch OJ, Toschi N, Torner L, Douglas AJ. 2003. No stress response of the hypothalamo-pituitary-adrenal axis in parturient rats: Lack of involvement of brain oxytocin. Endocrinology 144:2473–2479.

Ogawa S, Lozach J, Benner C, Pascual G, Tangirala RK, Westin S, Hoffmann A, Subramaniam S, David M, Rosenfeld MG, Glass CK. 2005. Molecular determinants of crosstalk between nuclear receptors and toll-like receptors. Cell 122:707–721.

Pacilio M, Migliaresi S, Meli R, Ambrosone L, Bigliardo B, Di Carlo R. 2001. Elevated bioactive prolactin levels in systemic lupus erythematosus—association with disease activity. J Rheumatol 28:2216–2221.

Peeva E, Zouali M. 2005. Spotlight on the role of hormonal factors in the emergence of autoreactive B-lymphocytes. Immunol Lett 101:123–143.

Peeva E, Grimaldi C, Spatz L, Diamond B. 2000. Bromocriptine restores tolerance in estrogen-treated mice. J Clin Invest 106:1373–1379.

Peeva E, Michael D, Cleary J, Rice J, Chen X, Diamond B. 2003. Prolactin modulates the naive B cell repertoire. J Clin Invest 111:275–283.

Petri M, Chan D. 1995. Longitudinal analysis of prolactin and disease activity in SLE. Arthritis Rheum 38:S220, (abstract)..

Rettori V, Gimeno MF, Karara A, Gonzalez MC, McCann SM. 1991. Interleukin 1 alpha inhibits prostaglandin E2 release to supress pulsatile release of luteinizing hormone but not follicle-stimulating hormone. Proc Natl Acad Sci USA 88:2763–2767.

Reuman PD. 2004. First reported pediatric case of systemic lupus erythematosus associated with prolactinoma. Arthritis Rheum 50:3616–3618.

Russell JA, Brunton PJ. 2006. Neuroactive steroids attenuate oxytocin stress responses in late pregnancy. Neuroscience 138:879–889.

Sajti E, van Meeteren N, Kavelaars A, van der Net J, Gispen WH, Heijnen C. 2004. Individual differences in behavior of inbred Lewis rats are associated with severity of joint destruction in adjuvant-induced arthritis. Brain Behav Immun 18:505–514.

Santora K, Rasa C, Visco D, Steinetz B, Bagnell C. 2005. Effects of relaxin in a model of rat adjuvant-induced artritis. Ann N Y Acad Sci 1041:481–485.

Schlaghecke R, Beuscher D, Kornely E, Specker C. 1994. Effects of glucocorticoids in rheumatoid arthritis. Diminished glucocorticoid receptors do not result in glucocorticoid resistance. Arthritis Rheum 37:1127–1131.

Shanks N, Moore PM, Perks P, Lightman SL. 1999. Alterations in hypothalamic-pituitary-adrenal function correlated with onset of murine SLE in MRL+/+ and lpr mice. Brain Behav Immunol 13:348–369.

Shim GJ, Warner M, Kim HJ, Andersson S, Liu L, Ekman J, et al. 2004. Aromatase-deficient mice spontaneously develop a lymphoproliferative autoimmune disease resembling Sjögren's syndrome. Proc Natl Acad Sci USA 101:12628–12633.

Spies CM, Schaumann DH, Berki T, Mayer K, Jakstadt M, Huscher D, et al. 2006. Membrane glucocorticoid receptors (mGCR) are down- regulated by glucocorticoids in patients with systemic lupus erythematosus and use a caveolin-1 independent expression pathway. Ann Rheum Dis. Jan 31; [Epub ahead of print].

Sternberg EM. 2006. Neural regulation of innate immunity: A coordinated nonspecific host response to pathogens. Nat Rev Immunol 6:318–328.

Sternberg EM, Hill JM, Chrousos GP, Kanilaris T, Listwak SJ, Wilder RL. 1989. Inflammatory mediator-induced hypothalamic-pituitary-adrenal axis activation is defective in streptococcal cell wall arthritis susceptible Lewis rats. Proc Natl Acad Sci USA 86:2374–2378.

Stevens A, Ray D, Alansari A, Hajeer A, Thomson W, Donn R, Ollier WE, Worthington J, Davis JR. 2001. Characterization of a prolactin gene polymorphism and its associations with systemic lupus erythematosus. Arthritis Rheum 44:2358–2366.

Straub RH, Cutolo M. 2001. Involvement of the hypothalamic—pituitary—adrenal/gonadal axis and the peripheral nervous system in rheumatoid arthritis: Viewpoint based on a systemic pathogenetic role. Arthritis Rheum 44:493–507.

Straub RH, Cutolo M. 2001. Involvement of the hypothalamic—pituitary—adrenal/gonadal axis and the peripheral nervous system in rheumatoid arthritis: Viewpoint based on a systemic pathogenetic role. Arthritis Rheum 44:493–507.

Straub RH, Besedovsky HO. 2003. Integrated evolutionary, immunological, and neuroendocrine framework for the pathogenesis of chronic disabling inflammatory diseases. FASEB J 17:2176–2183.

Straub RH, Struharova S, Scholmerich J, Harle P. 2002. No alterations of serum levels of adrenal and gonadal hormones in patients with ankylosing spondylitis. Clin Exp Rheumatol 20(6 Suppl 28):S52–S59.

Straub RH, Weidler C, Demmel B, Herrmann M, Kees F, Schmidt M, et al. 2004. Renal clearance and daily excretion of cortisol and adrenal androgens in patients with rheumatoid arthritis and systemic lupus erythematosus. Ann Rheum Dis 63:961–968.

Szyper-Kravitz M, Zandman-Goddard G, Lahita RG, Shoenfeld Y. 2005. The neuroendocrine-immune interactions in systemic lupus erythematosus: A basis for understanding disease pathogenesis and complexity. Rheum Dis Clin North Am 31:161–175.

Takizawa K, Kitani S, Takeuchi F, Yamamoto K. 2005. Enhanced expression of CD69 and CD25 antigen on human peripheral blood mononuclear cells by prolactin. Endocr J 52:635–641.

Tanaka H, Akama H, Ichikawa Y, Makino I, Homma M. 1992. Glucocorticoid receptor in patients with lupus nephritis: Relationship between receptor levels in mononuclear leukocytes and effect of glucocorticoid therapy. J Rheumatol 19:878–883.

Tangri S, Wegmann TG, Lin H, Raghupathy R. 1994. Maternal anti-placental reactivity in natural, immunologically-mediated fetal resorptions. J Immunol 152:4903–4911.

Tanriverdi F, Silveira LF, MacColl GS, Bouloux PM. 2003. The hypothalamic-pituitary-gonadal axis: Immune function and autoimmunity. J Endocrinol 176:293–304.

Tengstrand B, Carlstrom K, Fellander-Tsai L, Hafstrom I. 2003. Abnormal levels of serum dehydroepiandrosterone, estrone, and estradiol in men with rheumatoid arthritis: High correlation between serum estradiol and current degree of inflammation. J Rheumatol 30:2338–2343.

Tengstrand B, Carlstrom K, Hafstrom I. 2002. Bioavailable testosterone in men with rheumatoid arthritis-high frequency of hypogonadism. Rheumatology (Oxford) 41:285–289.

Tohyama CT, Yamakawa M, Murasawa A, Nakazono K, Ishikawa H. 2005. Localization of human glucocorticoid receptor in rheumatoid synovial tissue of the knee joint. Scand J Rheumatol 34:426–432.

Torner L, Toschi N, Nava G, Clapp C, Neumann ID. 2002. Increased hypothalamic expression of prolactin in lactation: Involvement in behavioural and neuroendocrine stress responses. Eur J Neurosci 15:1381–1389.

Turrin NP, Rivest S. 2004. Unraveling the molecular details involved in the intimate link between the immune and neuroendocrine systems. Exp Biol Med 229:996–1006.

Uthman I, Senecal JL. 1995. Onset of rheumatoid arthritis after surgical treatment of Cushing's disease. J Rheumatol 22(43):S158.

Valtysdottir ST, Wide L, Hallgren R. 2001. Low serum dehydroepiandrosterone sulfate in women with primary Sjögren's syndrome as an isolated sign of impaired HPA axis function. J Rheumatol 28:1259–1265.

Venkatesh J, Peeva E, Xu X, Diamond B. 2006. Cutting Edge: Hormonal milieu, not antigenic specificity, determines the mature phenotype of autoreactive B cells. J Immunol 176:3311–3314.

Vera-Lastra O, Jara LJ, Espinoza LR. 2002. Prolactin and autoimmunity. Autoimmun Rev 1:360–364.

Vera-Lastra O, Mendez C, Jara LJ, Cisneros M, Medina G, Ariza R, Espinoza LR. 2003. Correlation of prolactin serum concentrations with clinical activity and remission in patients with systemic lupus erythematosus. Effect of conventional treatment. J Rheumatol 30:2140–2146.

Vera-Lastra O, Jara LJ, Medina G, Rojas LJ, Velásquez F, Ariza R, et al. 2006. Functional hyperprolactinemia and hypophyseal microadenoma in systemic sclerosis. J Rheumatol 2006; 33:1108–1112.

Verthelyi D, Ahmed SA. 1994. 17 beta-estradiol, but not 5 alpha-dihydrotestosterone, augments antibodies to double-stranded deoxyribonucleic acid in nonautoimmune C57BL/6J mice. Endocrinology 135:2615–2622.

Walker SE, Jacobson JD. 2000. Roles of prolactin and gonadotropin-releasing hormone in rheumatic diseases. Rheum Dis Clin North Am 26:713–736.

Wang HC, Klein JR. 2001. Immune function of thyroid stimulating hormone and receptor. Crit Rev Immunol 21:323–337.

Wegmann TG, Lin H, Guilbert L, Mosmann TR. 1993. Bidirectional cytokine interactions in the maternal-fetal relationship: Is successful pregnancy a TH2 phenomenon? Immunol Today 14:353–356.

Weidler C, Härle P, Schedel J, Schmidt M, Schölmerich J, Straub RH. 2004. Patients with rheumatoid arthritis and systemic lupus erythematosus have increased renal excretion of mitogenic estrogens in relation to endogenous antiestrogens. J Rheumatol 31:489–494.

Weigent DA, Blalock JE. 1987. Interactions between the neuroendocrine and immune systems: Common hormones and receptors. Immunol Rev 100:79–108.

Wilder R. 1995. Neuroendocrine-immune system interactions and autoimmunity. Annu Rev Immunol 13:307.

Yu-Lee LY. 2002. Prolactin modulation of immune and inflammatory responses. Recent Prog Horm Res 57:435–455.

Zietz B, Reber T, Oertel M, Gluck T, Scholmerich J, Straub RH. 2000. Altered function of the hypothalamic stress axes in patients with moderately active systemic lupus erythematosus. II. Dissociation between androstenedione, cortisol, or dehydroepiandrosterone and interleukin 6 or tumor necrosis factor. J Rheumatol 27:911–918.

